# Patient‐Reported Outcome Measures for Severe Recurrent Bilateral Nasal Polyps: Psychometric Evaluation and Content Validity

**DOI:** 10.1002/oto2.84

**Published:** 2023-12-21

**Authors:** Adam Gater, Chloe Tolley, Rebecca Williams‐Hall, Claire Trennery, Helena Bradley, Mirko V. Sikirica, Linda Nelsen, Ana R. Sousa, Daniel J. Bratton, Robert Chan, Robyn von Maltzahn

**Affiliations:** ^1^ Patient‐Centered Outcomes, Adelphi Values Bollington Cheshire UK; ^2^ Value Evidence and Outcomes, GSK Philadelphia Pennsylvania USA; ^3^ Clinical Sciences, Respiratory, GSK Brentford Middlesex UK; ^4^ Clinical Statistics, GSK Brentford Middlesex UK; ^5^ Patient Centered Outcomes, Value Evidence and Outcomes, GSK Brentford Middlesex UK; ^6^ Present address: Market Access, Janssen Global Services, LLC

**Keywords:** chronic rhinosinusitis, disease severity, quality of life, visual analogue scale

## Abstract

**Objective:**

To date, no patient‐reported outcome measures have been specifically developed to assess pharmacological treatment effect in participants with severe chronic rhinosinusitis (CRS) with recurrent bilateral nasal polyps (NP). These studies aimed to assess (1) the psychometric properties and (2) content validity of Visual Analogue Scales (VAS) assessing NP symptom severity.

**Study Design:**

(1) Retrospective psychometric validation study using clinical trial data and (2) cross‐sectional qualitative patient interview study.

**Setting:**

(1) Multicentre trial; (2) real‐world.

**Methods:**

(1) Psychometric validation was performed using data from a randomized, double‐blind, placebo‐controlled, Phase II study (NCT01362244) investigating the effect of mepolizumab in 105 participants with severe, recurrent bilateral NP currently needing polypectomy surgery. (2) Content validity was explored through cognitive debriefing interviews in 27 adults with severe CRS with recurrent bilateral NP who had received NP surgery in the past 10 years (NCT03221192).

**Results:**

(1) Acceptable reliability, validity, and responsiveness were shown for individual VAS items, although the loss of smell VAS item performed poorly in several analyses, suggesting further evaluation of this item is needed. (2) All individual VAS items were well understood, considered relevant and were consistently interpreted by most participants, providing evidence for their content validity.

**Conclusion:**

These findings support the use of symptom VAS measures to evaluate disease experience and treatment effect in clinical trials of participants with severe CRS with recurrent bilateral NP.

Patients with Chronic Rhinosinusitis with nasal polyps (CRSwNP) experience a variety of symptoms, including nasal obstruction, presence of watery rhinorrhoea (anterior), postnasal drainage (posterior), reduced sense of smell, and facial pain/headache.^1^, ^2^ These symptoms substantially impact patient‐reported health‐related quality of life (HRQoL).^3^ Patients' perspectives of the impact of a medical condition provides a unique, clinically relevant view of the condition. Patient‐reported outcome (PRO) instruments are thus essential for supporting clinical trial endpoints during drug development.^4^

Current evidence supporting the use of PRO instruments to measure treatment benefit in patients with severe CRS with recurrent, bilateral NP is limited. The 2020 European position paper on rhinosinusitis and NP advocates use of Visual Analogue Scale (VAS) scores for assessing patient‐evaluated severity of individual symptoms and overall disease severity.^2^ A VAS measures a characteristic or attitude believed to range across a continuum of values. The ends are defined as the extreme limits of the parameter, measured along a line of fixed length (eg, 10 cm or 100 mm).^5^ While such instruments have demonstrated adequate measurement properties to assess symptom burden and experience in patients with chronic rhinosinusitis (CRS),^6^, ^7^ they have often been developed by physicians with little to no patient involvement, meaning they may not accurately reflect patients' experiences. Therefore, there is a need to ensure the items/symptoms evaluated, and the response scale are relevant to patients with severe CRS with recurrent bilateral NP.

Data are presented herein from 2 separate studies in adult participants with severe CRS with recurrent bilateral NP. A quantitative validation study evaluated psychometric properties of 4 individual VAS items (rhinorrhoea, mucus in throat, nasal blockage, and loss of smell), and a qualitative cognitive debriefing study explored content validity of 6 individual VAS items (nasal obstruction, nasal discharge, mucus in the throat, loss of smell, facial pain/pressure, and overall symptoms).

## Materials and Methods

### Psychometric Validation Study

#### Study design

Psychometric properties of individual VAS items were evaluated (GSK ID: HO‐16‐18043) using data from a randomized, double‐blind, placebo‐controlled, multicentre, Phase II study (NCT01362244) of mepolizumab in patients with severe, recurrent, bilateral NP, requiring polypectomy.^8^ Full details for the Phase II study have been previously published^8^; in brief, participants 18 to 70 years of age were randomized to receive mepolizumab 750 mg or placebo intravenously, once every 4 weeks for 24 weeks with concurrent low‐dose intranasal corticosteroids (InCS). Individual VAS item scores (rhinorrhoea, mucus in throat, nasal blockage, and loss of smell; on a scale of 0 [none] to 10 [as bad as you can imagine] cm) collected at baseline and Week 25, were used to facilitate psychometric evaluation of the measures. A VAS total score (combined score based on responses to all individual symptom items) was also calculated. All individual VAS items had a recall period of the past week.

#### Analysis

Psychometric validation of individual VAS items included: assessment of item performance; dimensionality analyses; correlational analyses (item‐to‐scale and inter‐item); internal consistency and test‐retest reliability; construct validity; and responsiveness (sensitivity to and interpretation of change). Details of the analysis methods employed are presented in Supplemental Table S1, available online. Analyses were performed using blinded data pooled across treatment groups.

### Cognitive Debriefing Study

#### Study design

A qualitative, cross‐sectional patient interview study was conducted in the United States and Germany (GSK ID: 206325; NCT03221192). Study details, including eligibility criteria, have been previously reported^9^; in brief, adults with severe CRS with recurrent bilateral NP were eligible for inclusion if they had received NP surgery in the past 10 years, were current candidates for polypectomy, had symptoms consistent with CRS, and were receiving InCS for management of their NP. Sample quotas ensured participants with a range of demographic, educational, and clinical characteristics were recruited in the United States and Germany.

Participants took part in 2‐hour, combined concept elicitation and cognitive debriefing telephone interviews conducted by trained qualitative interviewers (Adelphi Values, Bollington, Cheshire, UK). Concept elicitation findings have been previously published.^9^ During cognitive debriefing, participants completed 6 individual VAS items (nasal obstruction, nasal discharge, mucus in the throat, loss of smell, facial pain/pressure, and overall symptoms; with a scale of 0 (“no symptom”) to 100 (“as bad as you can imagine”) using a “think aloud” method, where participants were asked to voice their thoughts as they read the instructions and items and provided their response.^10^ These VAS items had been modified following their use in Phase II clinical study; changes included modification of the wording of item names, verbal descriptors, and response scale anchors to provide greater clarity and facilitate consistent interpretation, addition of facial pain/pressure VAS item to ensure comprehensive assessment of CRSwNP symptoms, and use of a recall period of the past 24 hours for all VAS items. Participants were then asked detailed follow‐up questions designed to explore understanding of the items, instructions, response options and recall period, and relevance to the patient's experience of severe CRS with recurrent bilateral NP. Participants also discussed what change in score for each item they considered as meaningful.

#### Analysis

All interviews were recorded, transcribed verbatim, and anonymized for the purpose of qualitative analysis. Interview transcripts were qualitatively analyzed using Atlas.ti software version 7 (ATLAS.ti Scientific Software Development GmbH) using a framework approach.

### Ethics Approval and Consent to Participate

Study NCT01362244 was conducted in accordance with the Declaration of Helsinki. Written informed consent was obtained from participants before the start of any procedures. As a multicentre study, the protocol was approved by the local ethics committee for each centre. Ethical approval for NCT03221192 (GSK study ID: 206325); was sought from Copernicus IRB, a centralized Independent Review Board based in the US (approval reference: ADE1‐17‐256) and the Freiburger Ethik‐Komission International board in Germany (approval reference: HO‐16‐17264). All participants provided written informed consent.

## Results

### Psychometric Validation Study

A total of 105 participants were randomized, received ≥1 dose of study drug, and included in the intent‐to‐treat population.^8^ Participants had mean (range) age of 50.2 (23‐70) years; 71.4% were women and 78.1% had a history of asthma. Mean (range) endoscopic nasal polyps (ENP) score in the worst nostril was 3.3 (3‐4).

#### Item and scale characteristics

Ceiling effects (≥15% of participants scoring 9‐10 cm) were present for 3 of 4 individual VAS items (rhinorrhoea, nasal blockage, loss of smell) at baseline and 2 of 4 items (nasal blockage and loss of smell) at Week 25 (Supplemental Table S2, available online). Floor effects (≥15% of participants scoring 0‐0.9 cm) were present for 3 of 4 items (rhinorrhoea, mucus in throat, nasal blockage) at Week 25. There was limited endorsement at the middle of the scale (3‐4.9 cm) across all items at both time points.

In the Confirmatory Factor Analysis (CFA), Comparative Fit Index (>0.999), root mean square error approximation (<0.001), and standardized root mean residual (0.020) fit statistics were supportive of a VAS total score. Factor loadings were strongest for the rhinorrhoea item (0.873), and weakest for loss of smell item (0.305). For item‐to‐scale correlations, 3 of 4 items correlated with VAS total score (Supplemental Table S3, available online). Only loss of smell correlated weakly with VAS total score (0.276). No items showed high inter‐item correlations suggesting no item redundancy (Supplemental Table S4, available online).

#### Reliability

Internal consistency of the VAS total score was *ɑ* = 0.68, slightly lower than the a priori threshold of *ɑ* ≥ 0.70 (Table 1). This reduced when the individual items rhinorrhoea, mucus in throat, and nasal blockage were removed (*ɑ* = 0.47, *ɑ* = 0.63, and *ɑ* = 0.56, respectively), suggesting these items contribute to reliability of the VAS total score. However, when the individual item loss of smell was removed, internal consistency increased to *ɑ* = 0.72, suggesting that this item may not be measuring the same underlying concept as the above items.

**Table 1:** Internal Consistency Reliability of the VAS Total Score at Baseline (*n* = 105)

	Cronbach's *α*
Internal consistency of VAS total score^a^	0.678
Internal consistency if item deleted^b^	
1. Rhinorrhoea	0.470
2. Mucus in throat	0.626
3. Nasal blockage	0.561
4. Loss of smell	*0.722*

Abbreviation: VAS, Visual Analogue Scale.

^a^ Cronbach's *α* >.70 is considered acceptable.

^b^ Value *italicized* if *α* increases when item deleted.

All 4 individual VAS items and total score met the pre‐specified threshold for adequate test‐retest reliability (intra‐class correlation [ICC] > 0.60) between baseline and Week 2 (ICC = 0.70‐0.79) (Table 2).

**Table 2:** Test‐Retest Reliability of the Individual VAS and VAS Total Scores Between Baseline and Week 2

			95% Confidence interval	
VAS	N^a^	Reliability (ICC)^b^	Lower	Upper
Rhinorrhoea	105	0.794	0.711	0.855
Mucus in throat	105	0.754	0.658	0.826
Nasal blockage	105	0.698	0.586	0.784
Loss of smell	105	0.697	0.584	0.783
VAS total score	105	0.717	0.610	0.798

Abbreviations: ICC, intra‐class correlation; VAS, Visual Analogue Scale.

^a^ For Individual Symptoms VAS, only participants that completed Individual Symptoms VAS and severity of condition VAS at baseline and Week 2 were included in the analysis.

^b^ ICC computed using Shrout‐Fleiss reliability: single score statistic using data from baseline and Week 2.

#### Construct validity

Convergent validity, demonstrated by Spearman correlations >0.40, was seen for some individual VAS items/total score and clinical parameters at study exit (Week 25 or early withdrawal due to lack of efficacy) (Table 3). All items moderately correlated with ENP score; nasal blockage, loss of smell, and VAS total scores correlated with total worst and total mean olfactory score, while peak nasal inspiratory flow only correlated with nasal blockage VAS score.

**Table 3:** Convergent and Discriminant Validity for VAS Scores and Concurrent Measures at Study Exit^a^

	Spearman correlations with VAS scores^b^, n = 74				
Concurrent measure	Rhinorrhoea	Mucus in throat	Nasal blockage	Loss of smell	VAS total score
ENP score^c^	0.587	0.486	0.699	0.436	0.643
Total worst olfactory score^d^	−0.391	−0.398	−0.446	−0.466	−0.523
Total mean olfactory score^d^	−0.381	−0.342	−0.446	−0.523	−0.526
Peak nasal inspiratory flow^e^	−0.395	−0.300	−0.428	−0.174	−0.364

For individual symptoms VAS, moderate to high correlations are underlined.

Abbreviations: ENP, endoscopic nasal polyps; VAS, Visual Analogue Scale.

^a^ At Week 25 or early withdrawal due to lack of efficacy.

^b^ VAS item scores range from 0 to 10 with higher scores associated with worse symptoms. Individual Symptoms VAS total score ranges from 0 to 40 with higher scores associated with worse symptoms.

^c^ ENP score ranges from 0 to 4 with higher scores associated with larger polyps.

^d^ Olfactory scores range from 0 to 12 with lower scores indicating worse sense of smell.

^e^ Peak nasal inspiratory flow in L/min with higher scores indicating greater lung capacity.

Individual VAS items demonstrated acceptable known groups validity based on eosinophil count (rhinorrhoea item), asthma comorbidity (rhinorrhoea and loss of smell items), aspirin sensitivity and aspirin sensitivity/asthma comorbidity (rhinorrhoea, nasal blockage and loss of smell items, VAS total score), and need for surgery (ENP score ≥3 OR ENP score =2 AND Severity of Condition VAS score >7, where the severity of condition VAS assessed overall NP symptoms [scale 0‐10]) at Week 25/study exit (all individual VAS items and total score) (Supplemental Table S5, available online).

#### Responsiveness

Overall, participants with a better response to therapy (improved vs stable or worsened) had greater median change in VAS scores (individual items and total score; *P* ≤ .001; Table 4). When response was defined as no longer requiring surgery (ENP and severity of condition VAS scores), median change from baseline to Week 25/study exit among responders ranged from −4.20 (loss of smell VAS) to −6.60 (nasal blockage VAS) (Supplemental Table S6, available online). Median change for each score was significantly greater (*P* < .005) among responders versus nonresponders. When response was defined as >2‐point change in severity of condition VAS scores between baseline and Week 25/study exit, median change among responders was slightly lower than for the primary responder estimates (Supplemental Table S6, available online).

**Table 4:** Change in VAS Score for Response to Therapy Groups (Worsened, Stable, or Improved) (n = 88)

Score/change group^a^	n	Mean change (SD)^b^	Median change^b^	*P* value^c^	SES^d^
Rhinorrhoea					
Worsened	24	−0.9 (3.8)	−0.0	<0.001	−0.30
Stable	43	−1.3 (3.1)	−0.4		−0.42
Improved	18	−5.1 (3.4)	−5.4		−1.66
Mucus in throat					
Worsened	24	−1.2 (3.4)	−0.2	0.001	−0.38
Stable	43	−0.8 (3.5)	−0.1		−0.28
Improved	18	−4.4 (2.9)	−4.7		−1.70
Nasal blockage					
Worsened	24	−2.1 (3.6)	−0.7	<0.001	−0.96
Stable	43	−2.1 (2.8)	−1.3		−0.88
Improved	18	−6.2 (2.5)	−6.5		−3.01
Loss of smell					
Worsened	24	−1.0 (3.2)	0.0	<0.001	−0.56
Stable	43	−1.5 (2.6)	0.0		−0.89
Improved	18	−4.9 (4.4)	−4.9		−1.80
VAS total score					
Worsened	24	−1.3 (3.2)	−0.1	<0.001	−0.69
Stable	43	−1.4 (2.6)	−0.6		−0.76
Improved	18	−5.1 (2.5)	−5.3		−2.95

Abbreviations: ENP, endoscopic nasal polyps; SD, standard deviation; SES, standardized effect size; VAS, Visual Analogue Scale.

^a^ Response to therapy was based on ENP score, severity of condition VAS, and corticosteroid use between baseline and study exit (Week 25 or early withdrawal due to lack of efficacy), as follows: “improved”—participants that no longer required surgery at the end of the study or who had a >2‐point decrease in their Severity of Condition VAS score between baseline and study exit; “stable”—participants that still required surgery at the end of the study or who had no change or a 1‐point change in either direction in their Severity of Condition VAS score between baseline and study exit; “worsened”—participants who had surgery, needed oral corticosteroids or had a >2‐point increase in their Severity of Condition VAS score between baseline and study exit.

^b^ VAS—higher scores associated with more severe symptoms.

^c^ *P*‐values are from a Kruskal‐Wallis test comparing differences between median scores between groups.

^d^ The SES is from Cohen's *d*, calculated as: [(Mean follow‐up) − (Mean baseline)]/SD at baseline.

### Cognitive Debriefing Study

Seventeen participants from the US and 10 from Germany took part in cognitive debriefing interviews. Participant demographics and baseline clinical characteristics have been reported previously.^9^ In brief, mean (range) age was 48.4 (20‐78) years; 18/27 participants were women. Over half of participants (16/27, 59.3%) had an overall VAS symptom score of 71 to 80 and most participants (19/27, 70.4%) had ENP total scores of 5 to 6.

**Figure 1:**
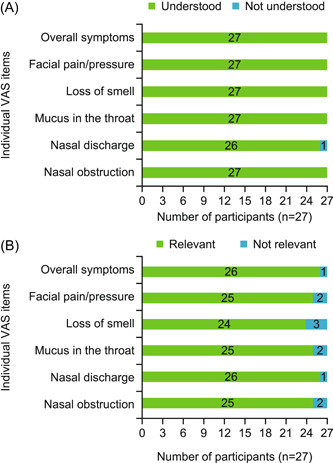
Overall understanding (A) and relevance (B) of Visual Analogue Scale items (n = 27).

All individual VAS items were understood by 96.3% to 100% (≥26/27) of participants (Figure 1A); 1 participant did not understand the nasal discharge VAS item, interpreting this symptom as postnasal drip. All individual VAS items were considered relevant by 88.9% to 96.3% (24/27‐26/27) of participants (Figure 1B). Participants described items as not relevant if they did not experience the symptom described as part of their condition. Participants did not report conceptual overlap for the VAS items.

Overall, the 24‐hour recall period was well understood by participants and shown to be relevant across all individual VAS items. Most participants reported their symptom severity varied across 24 hours, including 21/24 (87.5%) participants for nasal obstruction, 14/15 (93.3%) for nasal discharge, 18/22 (81.8%) for mucus in the throat, 12/20 (60.0%) for loss of smell, 9/12 (75.0%) for facial pain or pressure, and 18/21 (85.7%) for overall symptoms. Reported meaningful changes for each VAS item are shown in Supplemental Table S7, available online. Over 55% of participants reported as meaningful a reduction of ≤59% for nasal obstruction, nasal discharge and mucus in the throat, ≤69% for loss of smell and overall symptoms, and ≤79% for facial pain or pressure.

## Discussion

Overall, the psychometric validation study demonstrated acceptable reliability, validity, and responsiveness and supports the use of individual VAS to monitor patient‐reported treatment effects in clinical studies for treatment of severe CRS with recurrent bilateral NP requiring recurrent surgery. Nonetheless, findings indicated several ways in which individual VAS items and total symptom score may be optimized for use in this patient population.

Of the 4 individual VAS items assessed in the psychometric study (rhinorrhoea, mucus in throat, nasal blockage, and loss of smell), loss of smell had high ceiling effects at all time points, with the most severe level of symptoms reported in the 9 to 10 cm category and at the “true” ceiling of 10 cm. Thus, this item may be the most severe symptom experienced by participants. However, response categories in the middle of the scale were endorsed by few or no participants, suggesting that loss of smell is experienced as either present or absent, rather than on a continuum. Lack of variability and binary‐type response for this item likely impacted its poor performance in some analyses. For example, although CFA fit statistics suggested a total score may be appropriate for the individual VAS items, factor loadings were weakest for loss of smell, indicating that loss of smell differs from the other 3 individual VAS items and should perhaps be excluded from the VAS total score. Additionally, loss of smell VAS may have been affected by participants’ previous surgical history, as trauma to the olfactory epithelium, intranasal structural damage, and/or scarring due to previous surgeries can affect sense of smell.^11^, ^12^ Interestingly, a study of the correlation of total and individual symptoms VAS with the Sino‐Nasal Outcomes Test (SNOT)‐22 (a frequently used PRO measure of HRQoL), in patients with CRS also found that while these measures were well correlated, this association was weakest with the loss of smell VAS.^7^ Further evaluation of patients’ experience of loss of smell, and measurement properties of this item is therefore required.

Following use of the individual VAS items in the Phase II study and initial psychometric analyses, some items were modified before use in the cognitive debriefing study and a subsequent Phase III study. Changes included modifying wording of item names and verbal descriptors, adding a facial pain/pressure VAS item and use of a 24‐hour recall period. The cognitive debriefing interviews provided evidence of content validity of these updated individual VAS items. Response scales and instructions were relevant, well understood and consistently interpreted by participants. Additionally, the 24‐hour recall period for VAS items was well understood and considered appropriate. These modified VAS were used in the subsequent Phase III study and were found to have acceptable reliability, validity, and responsiveness,^13^ supporting findings from the current study.

The cognitive debriefing study also investigated the threshold for assessing meaningful change in the individual VAS items. Results suggested most participants would consider a reduction in VAS score of 59% to be meaningful for most symptoms. Whilst 59% is a high threshold for meaningful change, these findings are largely consistent with results of anchor‐based minimal important difference estimates in the psychometric validation study, as the median improvements in individual VAS items among participants no longer requiring surgery at study exit were similarly high. Moreover, in very severely symptomatic participants, large changes may be required for substantive benefit in HRQoL. Together, these results demonstrate that VAS scores can detect meaningful change in patients' experience of symptoms in response to treatment, supporting their use as clinical study endpoints for patients with severe CRS with recurrent bilateral NP requiring recurrent polypectomy surgery.

While this study provides important insights into the validation of PROs for NP, limitations should be considered. First, the Phase II study from which psychometric analysis data were extracted was not designed to assess the validity of individual symptoms VAS. The sample size was therefore relatively small for this type of analysis, especially in some subgroups for the known groups and ability to detect change analyses. The small sample size was compounded by the number of participants who withdrew early from the study; for the individual symptoms VAS 30/105 (29%) randomized participants withdrew from the study and 2 (2%) were lost to follow‐up before Week 25, leaving 74 (70%) participants with data at Week 25. Second, participants who withdrew were likely to have more severe disease (9/30 participants had nasal surgery after withdrawing), potentially biasing results and leading to overestimation of improvements from baseline and threshold for minimum clinically important difference. Third, the study population had limited diversity in age, gender, race and ethnicity, all of which may impact generalizability of findings. Both studies included a high percentage of female participants (71.4% and 66.7% in the psychometric validation and cognitive debriefing studies, respectively), which may influence outcomes, symptom perception, and reporting, since gender differences have been reported in the epidemiology, presentation, and treatment outcomes of CRS.^14^ Fourth, as all participants had severe NP at baseline, data variability was limited; this may have impacted some analyses by violating underpinning assumptions (eg, normally distributed data for factor analysis) and served as a confounding variable for other analyses (eg, potentially inflating test‐retest reliability and limiting convergent validity). Furthermore, as the Phase II study was not originally intended to evaluate psychometric properties of the instruments, there were some constraints on several analyses. For example, Patient Global Impression measures of severity and change are typically recommended as anchors (defining stability and change on measurement concepts) to support test‐retest reliability, ability to detect change, and interpretation of change. In the absence of these measures, alternatives (including severity of condition VAS scores and need for surgery) were employed to support these analyses. These criteria for improvement may have exceeded the threshold of improvement considered meaningful to participants, impacting findings. Further research to evaluate definitions of meaningful change would be valuable. Finally, individual VAS items used in the psychometric validation of the Phase II study data do not align precisely with VAS items used in the cognitive debriefing. Individual VAS items were updated following the Phase II study to improve content validity and the updated VAS items demonstrated strong psychometric properties in the subsequent Phase III study.^13^ We continue to consider optimization and validation of the VAS items to be an ongoing process.

## Conclusion

Our findings provide insights into the content validity of the individual symptoms VAS instrument in terms of respondent understanding and assessment of concepts most important to patients with severe CRS with recurrent bilateral NP requiring recurrent polypectomy surgery.

## Author Contributions

**Adam Gater**, contributed to the conception or design of the study, the acquisition of study data, and data analysis or interpretation; **Chloe Tolley**, contributed to the conception or design of the study, the acquisition of study data, and data analysis or interpretation; **Rebecca Williams‐Hall**, contributed to the conception or design of the study, the acquisition of study data, and data analysis or interpretation; **Claire Trennery**, contributed to the conception or design of the study, the acquisition of study data, and data analysis or interpretation; **Helena Bradley**, contributed to the conception or design of the study, the acquisition of study data, and data analysis or interpretation; **Mirko V. Sikirica**, contributed to the conception or design of the study, and data analysis or interpretation; **Linda Nelsen**, contributed to the conception or design of the study, and data analysis or interpretation; **Ana R. Sousa**, contributed to the conception or design of the study, and data analysis or interpretation; **Robert Chan**, contributed to the conception or design of the study, and data analysis or interpretation; **Robyn von Maltzahn**, contributed to the conception or design of the study, and data analysis or interpretation; **Daniel J. Bratton**, contributed to data analysis or interpretation. All authors contributed to the development of the manuscript, drafted the work, or revised it critically for important intellectual content, gave final approval of the version to be published, and agreed to be accountable for all aspects of the work.

## Disclosures

### Competing interests

Adam Gater, Rebecca Williams‐Hall, Claire Trennery, and Helena Bradley are employees of Adelphi Values, a health outcomes agency commissioned to conduct research by companies in the pharmaceuticals industry. Adelphi Values received funding from GSK to conduct the studies summarized in this manuscript. Chloe Tolley was an employee of Adelphi Values at the time of the study. Mirko V. Sikirica and Claire Trennery are former employees of GSK and hold stocks/shares in GSK. Linda Nelsen, Ana R. Sousa, Daniel J. Bratton, Robert Chan, and Robyn von Maltzahn are employees of GSK and hold stocks/shares in GSK.

### Funding source

These studies were funded by GSK (GSK ID HO‐16‐18043 and GSK ID 206325).

## Supporting information

Supplemental Material

## Data Availability

The data sets used and/or analyzed during the current study are available from the corresponding author on reasonable request.
