# Supplementary material for: Patient‐Reported Outcome Measures for Severe Recurrent Bilateral Nasal Polyps: Psychometric Evaluation and Content Validity
Source: OTO Open. 2023 Dec 21;7(4):e84. doi: 10.1002/oto2.84 (PMC10733778; doi:10.1002/oto2.84)
Supplement: Supplementary file 1 — Supplemental Material [file OTO2-7-e84-s001.docx]

# SUPPLEMENTARY MATERIALS

**Supplementary Tables**

**Table S1**. Psychometric analyses for VAS.

|  | Individual VAS items  ~~SNOT-22~~ |
| --- | --- |
| **Item and scale characteristics** | |
| Item performance | Distributional characteristics (N, mean, SD, and ceiling and floor effects) were used to assess item performance – ceiling effect refers to a high proportion of participants scoring the highest (worst) possible score for an item, while floor effect refers to a high proportion of participants scoring the lowest (best) score possible for an item. Items with >15% of participants scoring the highest or lowest scores were considered to have ceiling or floor effects.^1^ |
| Dimensionality analyses | CFA performed to determine if the four Individual Symptoms VAS items could be combined to form a total symptom score that would measure the latent variable of ‘nasal polyp symptom severity’, using data collected at baseline and overall model fit assessed using: CFI (values >0.95 indicate acceptable fit); RMSEA (values <0.06 are acceptable); SRMR (values <0.8 are acceptable)  EFA performed and overall model fit assessed using: CFI (values >0.95 indicate acceptable fit); RMSEA (values <0.06 are acceptable); SRMR (values <0.8 are acceptable). |
| Item relationships | Item-to-scale and inter-item correlational analyses conducted to explore the relationships between individual items and the scale scores derived at baseline. Spearman correlation coefficients were used as the data were not normally distributed.  For item-to-scale correlations, two criteria considered: item convergent validity (coefficients between each item and domain or total scores should be ≥0.37) and item discriminant validity (each item should have a higher coefficient with its own domain than any other).  For inter-item correlations, a correlation >0.80 indicated that items were highly correlated with each other. |
| *Reliability* | |
| Internal consistency reliability | Internal consistency of the VAS ~~i~~tem/total scores were evaluated using Cronbach’s α at study exit (Week 25 or early withdrawal due to lack of efficacy) with the commonly accepted threshold of >0.70 used to indicate that a set of items have enough in common to justify grouping together.^2^ |
| Test-retest reliability | Test-retest reliability at the item and total score level in participants with stable disease. ICC coefficients using the Shrout–Fleiss reliability were used; ICC scores >0.70 among participants categorised as stable (with reference to an external anchor) are generally considered evidence of acceptable test-retest reliability.^2, 3^ In this study, as stability between timepoints was not determined with reference to an external anchor, an ICC >0.60 was accepted as fair evidence of acceptable test-retest reliability. |
| *Construct validity* | |
| Convergent validity | Performed on endoscopic nasal polyposis (ENP) score, olfactory score and peak nasal inspiratory flow. Correlation coefficients (Pearson and Spearman) of 0.4–0.7 reflect moderate convergent validity. |
| Known-groups methods | Performed using baseline data in participants grouped according to the following baseline characteristics: blood eosinophil count (<300 cells/μL vs ≥300 cells/μL), presence of comorbid asthma (yes vs no); presence of comorbid aspirin sensitivity (yes vs no); and presence of comorbid asthma AND aspirin sensitivity (yes vs no). Subgroups were compared using t-tests, with p<0.05 considered statistically significant. |
| *Responsiveness* | |
| Ability to detect change | The extent to which scores changed with a change in disease severity was explored. ANOVA and relevant post hoc tests (e.g. Tukey’s honestly significant difference) were used to compare changes in scores between baseline and study exit. Participants were grouped according to response to therapy (‘worsened’ [had surgery, needed oral corticosteroids, or had a >2-point increase in Severity of Condition VAS score], ‘stable’ [still required surgery or had no change/1-point change in either direction in their Severity of Condition VAS score], ‘improved’ [no longer required surgery or had a >2-point decrease in their Severity of Condition VAS score]). Where the severity of condition VAS assessed how troublesome nasal polyposis symptoms were overall (scale 0–10). |
| Meaningful level of change | An anchor-based approach was conducted to establish a meaningful level of change that could be used as a responder threshold. Meaningful change was pre-specified as a participant no longer in need of surgery (ENP score ≤1 OR ENP score 2 AND Severity of Condition VAS score ≤7, at Week 25). Spearman correlations were conducted between Individual Symptoms VAS scores and a correlation of ≥0.4 was considered to warrant the anchor as useful. |

ANOVA, analysis of variance; CFA, confirmatory factor analysis; CFI, comparative fit index; ENP, endoscopic nasal polyps; ICC, intra-class correlation; RMSEA, root mean square error approximation; SD, standard deviation; SRMR, standardized root mean residual; VAS, Visual Analogue Scale; WRMR, weighted root mean residual.

1. Terwee CB, Bot SD, de Boer MR, et al. Quality criteria were proposed for measurement properties of health status questionnaires. *J Clin Epidemiol*. 2007;60(1):34-42. doi:10.1016/j.jclinepi.2006.03.012

2. Nunnally J. *Psychometric Theory*. 3rd ed. McGraw-Hill Inc; 1994.

3. Shrout PE, Fleiss JL. Intraclass correlations: uses in assessing rater reliability. *Psychol Bull*. 1979;86(2):420-8. doi:10.1037/0033-2909.86.2.420

**Table S2.** Item response distribution and floor and ceiling effects for the individual symptoms VAS at baseline (n=105) and Week 25 (n=74) in Study NCT01362244.

|  | Item response scale*^,†,‡^ | | | | | | | | | | | | | | | | | | | |
| --- | --- | --- | --- | --- | --- | --- | --- | --- | --- | --- | --- | --- | --- | --- | --- | --- | --- | --- | --- | --- |
| Individual VAS items | 0–0.9 cm | | 1–1.9 cm | | 2–2.9 cm | | 3–3.9 cm | | 4–4.9 cm | | 5–5.9 cm | | 6–6.9 cm | | 7–7.9 cm | | 8–8.9 cm | | 9–10.0 cm | |
|  | Baseline | Week 25 | Baseline | Week 25 | Baseline | Week 25 | Baseline | Week 25 | Baseline | Week 25 | Baseline | Week 25 | Baseline | Week 25 | Baseline | Week 25 | Baseline | Week 25 | Baseline | Week 25 |
| Rhinorrhoea | 7  (6.7%) | **28**  **(37.8%)** | 8  (7.6%) | 10  (13.5%) | 7  (6.7%) | *2*  *(2.7%)* | *2*  *(1.9%)* | *3*  *(4.1%)* | 7  (6.7%) | *3*  *(4.1%)* | 7  (6.7%) | *1*  *(1.4%)* | 12  (11.4%) | 6  (8.1%) | 16  (15.2%) | 5  (6.8%) | 17  (16.2%) | *4*  *(5.4%)* | **22**  **(21.0%)** | 11  (14.9%) |
| Mucus in throat | 11  (10.5%) | **24**  **(32.4%)** | *2*  *(1.9%)* | *4*  *(5.4%)* | 5  (4.8%) | 6  (8.1%) | 5  (4.8%) | 6  (8.1%) | *3*  *(2.9%)* | *3*  *(4.1%)* | 13  (12.4%) | *3*  *(4.1%)* | 13  (12.4%) | 6  (8.1%) | 17  (16.2%) | 8  (10.8%) | 21  (20.0%) | 5  (6.8%) | 15  (14.3%) | 8  (10.8%) |
| Nasal blockage | *1*  *(1.0%)* | **17**  **(23.0%)** | *2*  *(1.9%)* | 10  (13.5%) | *3*  *(2.9%)* | 7  (9.5%) | *2*  *(1.9%)* | *2*  *(2.7%)* | *1*  *(1.0%)* | *3*  *(4.1%)* | 7  (6.7%) | 7  (9.5%) | 6  (5.7%) | *4*  *(5.4%)* | 16  (15.2%) | *3*  *(4.1%)* | 21  (20.0%) | 6  (8.1%) | **46**  **(43.8%)** | **14**  **(18.9%)** |
| Loss of smell | *1*  *(1.0%)* | 11  (14.9%) | - | 6  (8.1%) | - | *2*  *(2.7%)* | *4*  *(3.8%)* | *1*  *(1.4%)* | *3*  *(2.9%)* | *1*  *(1.4%)* | - | *4*  *(5.4%)* | *1*  *(1.0%)* | *2*  *(2.7%)* | *4*  *(3.8%)* | *4*  *(5.4%)* | 5  (4.8%) | *1*  *(1.4%)* | **87**  **(82.9%)** | **41**  **(55.4%)** |

*0–9 mm =not troublesome, 9.0–10 cm=worst possible troublesome.

^†^Percentage calculated out of the number of participants completing the item at each time point; ^‡^any response options for which there is a limited endorsement (n<5) are *italicized*. Floor or ceiling effects (≥15% participants scoring at floor or ceiling) are highlighted in **bold**.

VAS, Visual Analogue Scale.

**Table S3**. Item-to scale correlations for the Individual Symptoms VAS items at baseline (n=105) in Study NCT01362244.

| Individual VAS items | Mean (SD) | Spearman item-to-scale correlations with  VAS total score |
| --- | --- | --- |
| Rhinorrhoea | 6.26 (2.94) | 0.607 |
| Mucus in throat | 6.20 (2.81) | 0.423 |
| Nasal blockage | 8.01 (2.15) | 0.431 |
| Loss of smell | 9.13 (1.79) | *0.276* |

Correlation coefficient ≥0.37 indicates item convergent validity; Italicised correlations did not meet this threshold.

SD, standard deviation; VAS, Visual Analogue Scale.

**Table S4.** Spearman inter-item correlations for Individual Symptoms VAS at baseline (n=105) in Study NCT01362244.

| Individual VAS items* | Rhinorrhoea | Mucus in throat | Nasal blockage | Loss of smell |
| --- | --- | --- | --- | --- |
| Rhinorrhoea | 1.000 | - | - | - |
| Mucus in throat | 0.434 | 1.000 | - | - |
| Nasal blockage | 0.539 | 0.207 | 1.000 | - |
| Loss of smell | 0.330 | 0.099 | −0.062 | 1.000 |

*Participants responded on a 0–10 cm VAS scale, with 0 representing ‘not troublesome’ and 10 representing ‘worst possible troublesome’.

VAS, Visual Analogue Scale.

**Table S5**. Construct validity using the known-groups method for individual VAS items and VAS total score^†^ (n=105).

|  |  | **Mean (SD)/median^‡^** | | | | |
| --- | --- | --- | --- | --- | --- | --- |
|  |  | **Rhinorrhoea** | **Mucus in throat** | **Nasal blockage** | **Loss of smell** | **VAS total score** |
| Eosinophil count | ≥300 cells/µL  (n=72) | **6.32 (2.97)**  **7.1*** | 6.00 (2.94)  6.8 | 7.91 (2.40)  8.8 | 8.89 (2.45)  9.7 | 7.34 (1.81)  7.9 |
|  | <300 cells/µL  (n=18) | **4.93 (3.10)**  **4.8*** | 6.29 (2.83)  7.5 | 8.09 (1.72)  8.3 | 9.12 (1.62)  9.8 | 7.05 (1.82)  7.6 |
| Participant has asthma | Yes  (n=82) | **6.67 (2.67)**  **7.3*** | 6.30 (2.67)  7.0 | 8.18 (1.97)  8.8 | **9.26 (1.59)**  **9.9**** | 7.60 (1.55)  8.0 |
|  | No  (n=23) | **4.80 (3.41)**  **4.6*** | 5.87 (3.31)  7.6 | 7.42 (2.64)  8.1 | **8.65 (2.34)**  **9.6**** | 6.69 (2.25)  6.5 |
| Participant has aspirin sensitivity | Yes  (n=46) | **7.21 (2.40)**  **7.8**** | 6.52 (2.57)  7.4 | **8.70 (1.09)**  **9.1*** | **9.31 (1.64)**  **9.9*** | **7.93 (1.31)**  **8.2**** |
|  | No  (n=56) | **5.38 (3.09)**  **5.8**** | 5.89 (3.01)  6.8 | **7.44 (2.60)**  **8.2*** | **8.94 (1.94)**  **9.6*** | **6.91 (1.94)**  **7.4**** |
| Participant has asthma and aspirin sensitivity | Yes  (n=42) | **7.33 (2.27)**  **8.0**** | 6.53 (2.46)  7.4 | **8.72 (1.00)**  **9.1*** | **9.30 (1.71)**  **9.9*** | **7.97 (1.18)**  **8.2**** |
|  | No  (n=60) | **5.42 (3.10)**  **6.0**** | 5.93 (3.05)  6.8 | **7.50 (2.56)**  **8.3*** | **8.98 (1.88)**  **9.6*** | **6.96 (1.97)**  **7.4**** |
| Requirement for surgery^§^ | Yes  (n=51) | **4.62 (3.77)**  **4.7***** | **4.84 (3.57)**  **5.3**** | **5.67 (3.46)**  **5.7***** | **7.85 (3.25)**  **9.7**** | **5.75 (2.98)**  **6.1**** |
|  | No  (n=21) | **1.33 (1.75)**  **0.7***** | **2.12 (2.24)**  **1.8**** | **1.58 (1.89)**  **0.8***** | **4.15 (4.12)**  **1.5**** | **2.29 (1.91)**  **2.1***** |

*p<0.05; **p<0.01; ***p<0.001.
^†^Construct validity was assessed at baseline by eosinophil count, asthma comorbidity, aspirin sensitivity, and asthma comorbidity and aspirin sensitivity at baseline, and at Week 25 by requirement for surgery at Week 25/study exit (Week 25 or early withdrawal due to lack of efficacy); ^‡^Non-parametric p-values are from a Mann–Whitney U test comparing differences between median scores between groups; ^§^defined using Severity of Condition VAS/ENP scores at study exit.

ENP, endoscopicnasal polyps; SD, standard deviation; VAS, Visual Analogue Scale.

**Table S6**. Anchor-based MID estimates of the individual VAS scores and VAS total score at Week 25/study exit (Week 25 or early withdrawal due to lack of efficacy) (N=74).

|  |  | No longer requiring surgery according to nasal polyps/severity of condition VAS response^†^ | | | | | | No longer requiring surgery according to severity of condition VAS response^‡^ | | | | | |
| --- | --- | --- | --- | --- | --- | --- | --- | --- | --- | --- | --- | --- | --- |
| VAS* | Responder group | Baseline median | Study exit median | N | Median change | Within group  p-value^§^ | Between groups  p-value^¶^ | Baseline median | Study exit median | N | Median change | Within group  p-value^§^ | Between groups  p-value^¶^ |
| Rhinorrhoea | Responder | 7.40 | 0.70 | 21 | −5.50 | <0.001 | <0.001 | 6.70 | 0.90 | 45 | −4.10 | <0.001 | <0.001 |
|  | Non-responder | 6.50 | 4.70 | 51 | −0.40 | 0.028 |  | 7.25 | 8.00 | 28 | 0.50 | 0.005 |  |
| Mucus in throat | Responder | 7.50 | 1.80 | 21 | −5.20 | <0.001 | <0.001 | 7.40 | 1.80 | 45 | −3.60 | <0.001 | <0.001 |
|  | Non-responder | 6.30 | 5.30 | 51 | −0.10 | 0.308 |  | 6.10 | 7.55 | 28 | 0.50 | 0.038 |  |
| Nasal blockage | Responder | 8.50 | 0.80 | 21 | −6.60 | <0.001 | <0.001 | 8.40 | 1.60 | 45 | −5.10 | <0.001 | <0.001 |
|  | Non-responder | 8.60 | 5.70 | 51 | −1.40 | <0.001 |  | 8.90 | 8.95 | 28 | 0.00 | 0.638 |  |
| Loss of smell | Responder | 9.90 | 1.50 | 21 | −4.20 | <0.001 | 0.003 | 9.80 | 5.00 | 45 | −2.50 | <0.001 | <0.001 |
|  | Non-responder | 9.70 | 9.70 | 51 | 0.00 | 0.003 |  | 9.70 | 9.80 | 28 | 0.00 | 0.259 |  |
| VAS total score | Responder | 7.60 | 2.05 | 21 | −5.43 | <0.001 | <0.001 | 7.53 | 2.73 | 45 | −4.05 | <0.001 | <0.001 |
|  | Non-responder | 7.50 | 6.08 | 51 | −0.62 | 0.003 |  | 7.58 | 8.24 | 28 | 0.39 | 0.032 |  |

*VAS item scores range from 0–10 with higher scores associated with worse symptoms. Individual Symptoms VAS total score ranges from 0–40 with higher scores associated with worse symptoms; ^†^responders were defined as those participants at Week 25/study exit who had an ENP score of <2 OR an ENP score of 2 AND a Severity of Condition VAS score of ≤7; ^‡^responders were defined as those participants with a >2-point change on the Severity of condition VAS between baseline and Week 25/study exit; ^§^the within group p-value is from a Wilcoxon Signed Rank test on change scores at each level of ENP score/Severity of Condition VAS response; ^¶^The between group p-value is from a Kruskal-Wallis testing distributional shift in change scores between ENP score/Severity of Condition VAS response groups.

ENP, eosinophilic nasal polyposis; VAS, Visual Analogue Scale.

**Table S7**. Reported meaningful changes during cognitive debriefing for the individual VAS items in Study NCT01362244.

| Reduction (%) | VAS item, n (%) | | | | | |
| --- | --- | --- | --- | --- | --- | --- |
|  | Nasal obstruction (n=17) | Nasal discharge (n=19) | Mucus in throat (n=19) | Loss of smell (n=18) | Facial pain or pressure (n=21) | Overall symptoms (n=21) |
| **100** | 2 (11.8) | 3 (15.8) | 2 (10.5) | 2 (11.1) | 5 (23.9) | 1 (4.8) |
| **90–99** | 0 | 0 | 1 (5.2) | 0 | 0 | 1 (4.8) |
| **80–89** | 1 (5.9) | 2 (10.5) | 0 (0) | 5 (27.7) | 3 (14.3) | 1 (4.8) |
| **70–79** | 1 (5.9) | 0 (0) | 2 (10.5) | 1 (5.5) | 4 (19.0) | 5 (23.9) |
| **60–69** | 2 (11.8) | 2 (10.5) | 1 (5.2) | 3 (16.6) | 0 | 3 (14.3) |
| **50–59** | 6 (35.3) | 4 (21.1) | 5 (26.3) | 2 (11.1) | 4 (19.0) | 1 (4.8) |
| **40–49** | 2 (11.8) | 3 (15.8) | 3 (15.8) | 4 (22.2) | 1 (4.8) | 0 |
| **30–39** | 2 (11.8) | 1 (5.2) | 4 (21.1) | 1 (5.5) | 1 (4.8) | 4 (19.0) |
| **20–29** | 0 | 2 (10.5) | 1 (5.2) | 0 | 3 (14.3) | 3 (14.3) |
| **10–19** | 0 | 2 (10.5) | 0 | 0 | 0 | 2 (9.5) |

VAS, Visual Analogue Scale.
